# Better survival in right-sided versus left-sided stage I - III colon cancer patients

**DOI:** 10.1186/s12885-016-2412-0

**Published:** 2016-07-28

**Authors:** Rene Warschkow, Michael C. Sulz, Lukas Marti, Ignazio Tarantino, Bruno M. Schmied, Thomas Cerny, Ulrich Güller

**Affiliations:** 1 Department of Surgery, Kantonsspital St. Gallen, 9007 St. Gallen, Switzerland; 2 Institute of Medical Biometry and Informatics, University of Heidelberg, 69120 Heidelberg, Germany; 3 Division of Gastroenterology and Hepatology, Kantonsspital St. Gallen, 9007 St. Gallen, Switzerland; 4 Department of General, Abdominal and Transplant Surgery, University of Heidelberg, 69120 Heidelberg, Germany; 5 Division of Medical Oncology & Hematology, Kantonsspital St. Gallen, 9007 St. Gallen, Switzerland; 6 University Clinic for Visceral Surgery and Medicine, University Hospital Berne, 3010 Berne, Switzerland

**Keywords:** Colon cancer, Right-sided, Left-sided, Survival, SEER

## Abstract

**Background:**

The distinction between right-sided and left-sided colon cancer has recently received considerable attention due to differences regarding underlying genetic mutations. There is an ongoing debate if right- versus left-sided tumor location itself represents an independent prognostic factor. We aimed to investigate this question by using propensity score matching.

**Methods:**

Patients with resected, stage I - III colon cancer were identified from the Surveillance, Epidemiology, and End Results (SEER) database (2004–2012). Both univariable and multivariable Cox regression as well as propensity score matching were used.

**Results:**

Overall, 91,416 patients (51,937 [56.8 %] with right-sided, 39,479 [43.2 %] with left-sided colon cancer; median follow-up 38 months) were eligible. In univariable analysis, patients with right-sided cancer had worse overall (hazard ratio [HR] = 1.32, 95 % CI:1.29–1.36, *P* < 0.001) and cancer-specific survival (HR = 1.26, 95 % CI:1.21–1.30, *P* < 0.001) compared to patients with left-sided cancer. After propensity score matching, the prognosis of right-sided carcinomas was better regarding overall (HR = 0.92, 95 % CI: 0.89 − 0.94, *P* < 0.001) and cancer-specific survival (HR = 0.90, 95 % CI:0.87 − 0.93, *P* < 0.001). In stage I and II, the prognosis of right-sided cancer was better for overall (HR = 0.89, 95 % CI:0.84–0.94 and HR = 0.85, 95 % CI:0.81–0.89) and cancer-specific survival (HR = 0.71, 95 % CI:0.64 − 0.79 and HR = 0.75, 95 % CI:0.70–0.80). Right- and left-sided colon cancer had a similar prognosis for stage III (overall: HR = 0.99, 95 % CI:0.95–1.03 and cancer-specific: HR = 1.04, 95 % CI:0.99–1.09).

**Conclusions:**

This population-based analysis on stage I - III colon cancer provides evidence that the prognosis of localized right-sided colon cancer is better compared to left-sided colon cancer. This questions the paradigm from previous research claiming a worse survival in right-sided colon cancer patients.

## Background

Colorectal cancer is one of the most commonly diagnosed cancers worldwide. The incidence is estimated to be 1.2 million per annum, and more than 600 000 patients die from this cancer every year [1, 2], hence representing a relevant public health problem. Over the past years, the distinction between right-sided and left-sided colon cancer has been brought into focus due to several reasons: Recent studies have revealed an increased frequency of right-sided colon cancer over the past decade [3, 4], which prompted the investigation for potential reasons of variation by anatomic sites. As shown by a recent systematic review, many publications pointed out several differences between right-sided and left-sided colon cancer regarding epidemiology, clinical presentation, pathology, and genetic mutations [5]. It has been shown that patients with right-sided colon cancer were older, more often female, had more advanced tumor stages, increased tumor sizes, more often poorly differentiated tumors, and different molecular biological tumor patterns [4, 6–13].

Data regarding prognosis in right-sided versus left-sided colon cancer are conflicting, and it remains a matter of great debate whether tumor location itself has a significant prognostic impact. The majority of studies demonstrated a poorer survival in right-sided compared to left-sided colon cancer [14–18]. In contrast to those data, Weiss et al. [19] found no overall difference in 5-year mortality between right- and left-sided colon cancer after adjusting for various variables.

The objective of the present population analysis of 91,416 colon cancer patients from the Surveillance, Epidemiology, and End Results (SEER) Program was to compare overall and cancer-specific survival between two large, virtually identical groups of patients with right- and left-sided colon cancer using propensity-score matching.

## Methods

### Cohort definition: surveillance, epidemiology, and end results

**Fig. 1:**
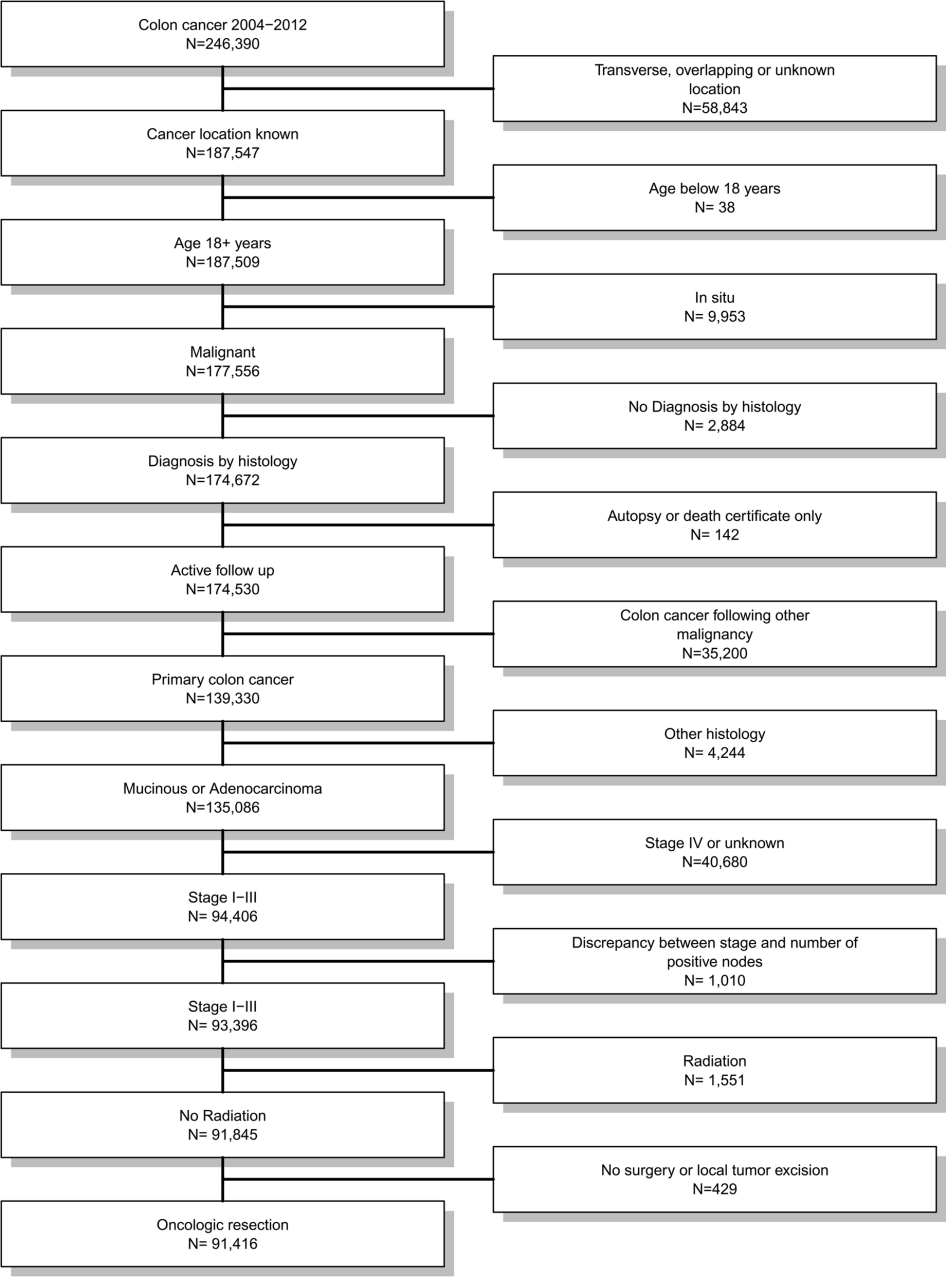
Flow chart of patients’ cohort definition. Data from the Surveillance, Epidemiology, and End Results (SEER) Program of the National Cancer Institute in the United States, covering approximately 28 % of cancer cases in the United States were used for the present population-based analysis. Primary cancer site and histology were coded according to criteria in the third edition of the International Classification of Diseases for Oncology (ICD-O-3) and used to identify 246,390 patients with colon cancer diagnosed between 2004 and 2012. The figure shows the selection process leaving 51,937 patients with right-sided and 39,479 patients with left-sided colon cancer for analysis

Data from the Surveillance, Epidemiology, and End Results (SEER) Program of the National Cancer Institute in the United States, covering approximately 28 % of cancer cases in the United States were used for the present population-based analysis [20]. SEER data were collected and reported using data items and codes as documented by the North American Association of Central Cancer Registries (NAACCR) [21]. Primary cancer site and histology were coded according to criteria in the third edition of the International Classification of Diseases for Oncology (ICD-O-3) and used to identify 246,390 patients with colon cancer diagnosed between 2004 and 2012 [22]. Patients with cancer of the cecum and ascending colon were accounted for right-sided colon cancer and patients with cancer of the descending or sigmoid colon were accounted for left-sided colon cancer. Patients with cancer on other or unknown location of the cancer were excluded. Figure 1 depicts the selection process leaving 51,937 patients with right-sided and 39,479 patients with left-sided colon cancer for analysis.

### Statistical analysis

Statistical analyses were performed using the R statistical software (www.r-project.org). A two-sided *p*-value <0.05 was considered statistically significant. Chi-Square statistics were used to analyze proportions. After descriptive analysis, the impact of location of colon cancer (right-sided versus left-sided) on overall and cancer-specific survival was assessed by Cox regression analysis with and without risk-adjustment for tumor stage, T-stage, retrieval and positivity of regional lymph nodes, grading, histology, preoperative serum carcinoembryonic antigen (CEA), type of operation, year of diagnosis, age, gender, ethnicity, and marital status (risk set). Additionally, a backward variable selection procedure from the full Cox regression model based on the Akaike’s information criterion was performed. The proportional hazard assumption was tested by scaled Schoenfeld residuals and by inspection of the hazard ratio (HR) plots [23]. The imbalances regarding prognostic factors between patients with right-sided and left-sided colon cancer were assessed by multivariable logistic regression. To further adjust for these differences in baseline characteristics and hence further minimizing bias, a propensity score matched analysis was performed as a further statistical method for adjustment [24–26] using the “MatchIt” R package [27]. The propensity score matching was performed as a full bipartite weighting matching procedure [28] with stratification for year of diagnosis and tumor stage. In this procedure, each patient with left-sided colon cancer was matched to all possible patients with right-sided colon cancer, forming subclasses and assigning weights such that within each subclass both groups have similar covariate values. Patients with right-sided colon cancer without a counterpart among the patients with left-sided colon cancer and vice versa were excluded from this analysis. A multivariable logistic regression conditional on the subgroups obtained by the matching procedure was performed to assess the persisting bias. Thereafter, overall and cancer-specific survival in patients with right-sided and left-sided colon cancer were assessed in a Cox regression analysis using the weights and strata obtained by the matching propensity score analysis. Finally, the propensity score matching procedure was repeated separately for stage I, II, and III colon cancer.

## Results

### Patient characteristics

For the present investigation 91,416 of 246,390 patients diagnosed with colon cancer between 2004 and 2012 were included (Fig. 1). The mean follow up was 42.3 ± 30.5 months and the median follow-up was 38 months (IQR: 16 to 66 months). At the end of follow-up period, 66,082 (72.3 %) patients were alive, 13,507 (14.8 %) died from colon cancer and 11,827 (12.9 %) died due to other reasons.

Overall, 51,937 (56.8 %) patients had right-sided colon cancer including 27,548 (30.1 %) patients with cancer of the cecum and 24,389 (26.7 %) patients with cancer of the ascending colon. Left-sided colon cancer was diagnosed in 39,479 (43.2 %) patients including 7039 (7.7 %) patients with cancer of the descending colon and 32,440 (35.5 %) patients with cancer of the sigmoid colon. Table 1 compares the patients’ characteristics for the two groups indicating relevant imbalances for all patients’ characteristics except for CEA-stage (*P* = 0.187). Patients with right-sided colon cancer had more advanced tumor stages, higher T-stages, had more regional lymph nodes retrieved, a higher grading, more often mucinous carcinomas, were more often Caucasian or African-American, were older, less often married, and were more often female (all *P* < 0.001).

**Table 1:** Patient characteristics

		Patient characteristics			
		Total	Right-sided carcinoma	Left-sided carcinoma	P^a^
		*N* = 91,416	*N* = 51,937	*N* = 39,479	
Tumor stage	Stage I	25,482 (27.9 %)	13,864 (26.7 %)	11,618 (29.4 %)	<0.001
(AJCC 6^th^ ed.)	Stage II	33,323 (36.5 %)	19,965 (38.4 %)	13,358 (33.8 %)	
	Stage III	32,611 (35.7 %)	18,108 (34.9 %)	14,503 (36.7 %)	
T-stage	T1	14,110 (15.4 %)	6415 (12.4 %)	7695 (19.5 %)	<0.001
	T2	15,873 (17.4 %)	9580 (18.4 %)	6293 (15.9 %)	
	T3	51,416 (56.2 %)	29,980 (57.7 %)	21,436 (54.3 %)	
	T4	10,017 (11.0 %)	5962 (11.5 %)	4055 (10.3 %)	
Number of positive	0	58,805 (64.3 %)	33,829 (65.1 %)	24,976 (63.3 %)	<0.001
regional lymph nodes	1	10,557 (11.5 %)	5627 (10.8 %)	4930 (12.5 %)	
	2–3	10,531 (11.5 %)	5672 (10.9 %)	4859 (12.3 %)	
	4–6	6560 (7.2 %)	3692 (7.1 %)	2868 (7.3 %)	
	7+	4963 (5.4 %)	3117 (6.0 %)	1846 (4.7 %)	
Number of retrieved	<12 lymph nodes	24,256 (26.5 %)	10,243 (19.7 %)	14,013 (35.5 %)	<0.001
regional lymph nodes	12–16 lymph nodes	26,123 (28.6 %)	14,917 (28.7 %)	11,206 (28.4 %)	
	17+ lymph nodes	41,037 (44.9 %)	26,777 (51.6 %)	14,260 (36.1 %)	
Grading	G1	8568 (9.4 %)	4721 (9.1 %)	3847 (9.7 %)	<0.001
	G2	64,221 (70.3 %)	34,812 (67.0 %)	29,409 (74.5 %)	
	G3	14,228 (15.6 %)	9886 (19.0 %)	4342 (11.0 %)	
	G4	1490 (1.6 %)	1099 (2.1 %)	391 (1.0 %)	
	Unknown	2909 (3.2 %)	1419 (2.7 %)	1490 (3.8 %)	
Histology	Adenocarcinoma	64,760 (70.8 %)	35,637 (68.6 %)	29,123 (73.8 %)	<0.001
	Adeno-Ca in adenomatous polyp	6791 (7.4 %)	2885 (5.6 %)	3906 (9.9 %)	
	Adeno-Ca in villous adenoma	2646 (2.9 %)	1721 (3.3 %)	925 (2.3 %)	
	Adeno-Ca in tubulovillous adenoma	8142 (8.9 %)	4897 (9.4 %)	3245 (8.2 %)	
	Mucinous carcinoma	9077 (9.9 %)	6797 (13.1 %)	2280 (5.8 %)	
CEA	C0-stage	32,999 (36.1 %)	18,679 (36.0 %)	14,320 (36.3 %)	0.185
	C1-stage	17,232 (18.9 %)	9896 (19.1 %)	7336 (18.6 %)	
	Unknown/Borderline	41,185 (45.1 %)	23,362 (45.0 %)	17,823 (45.1 %)	
Operation	Segmental resection	36,201 (39.6 %)	7750 (14.9 %)	28,451 (72.1 %)	<0.001
	Hemi/subtotal colectomy	54,241 (59.3 %)	43,777 (84.3 %)	10,464 (26.5 %)	
	Other	974 (1.1 %)	410 (0.8 %)	564 (1.4 %)	
Year	2004 to 2006	31,514 (34.5 %)	17,703 (34.1 %)	13,811 (35.0 %)	0.005
	2007 to 2009	31,314 (34.3 %)	17,798 (34.3 %)	13,516 (34.2 %)	
	2010 to 2012	28,588 (31.3 %)	16,436 (31.6 %)	12,152 (30.8 %)	
Age	Up to 49 years	8015 (8.8 %)	3175 (6.1 %)	4840 (12.3 %)	<0.001
	50 to 64 years	26,347 (28.8 %)	12,344 (23.8 %)	14,003 (35.5 %)	
	65 to 79 years	35,693 (39.0 %)	21,435 (41.3 %)	14,258 (36.1 %)	
	80+ years	21,361 (23.4 %)	14,983 (28.8 %)	6378 (16.2 %)	
Gender	Male	43,981 (48.1 %)	23,053 (44.4 %)	20,928 (53.0 %)	<0.001
	Female	47,435 (51.9 %)	28,884 (55.6 %)	18,551 (47.0 %)	
Ethnicity	Caucasian	72,937 (79.8 %)	42,296 (81.4 %)	30,641 (77.6 %)	<0.001
	African-American	10,572 (11.6 %)	6286 (12.1 %)	4286 (10.9 %)	
	Other/Unknown	7907 (8.6 %)	3355 (6.5 %)	4552 (11.5 %)	
Marital status	Married	49,901 (54.6 %)	27,219 (52.4 %)	22,682 (57.5 %)	<0.001
	Single	11,581 (12.7 %)	6120 (11.8 %)	5461 (13.8 %)	
	Widowed	17,770 (19.4 %)	11,835 (22.8 %)	5935 (15.0 %)	
	Other/Unknown	12,164 (13.3 %)	6763 (13.0 %)	5401 (13.7 %)	

All data shown as n (%)

AJCC, American Joint Committee on Cancer, *T-stage* tumor stage, *CEA* Carcinoembryonic antigen

^a^Chi-Square test

### Impact of cancer location on survival

**Fig. 2:**
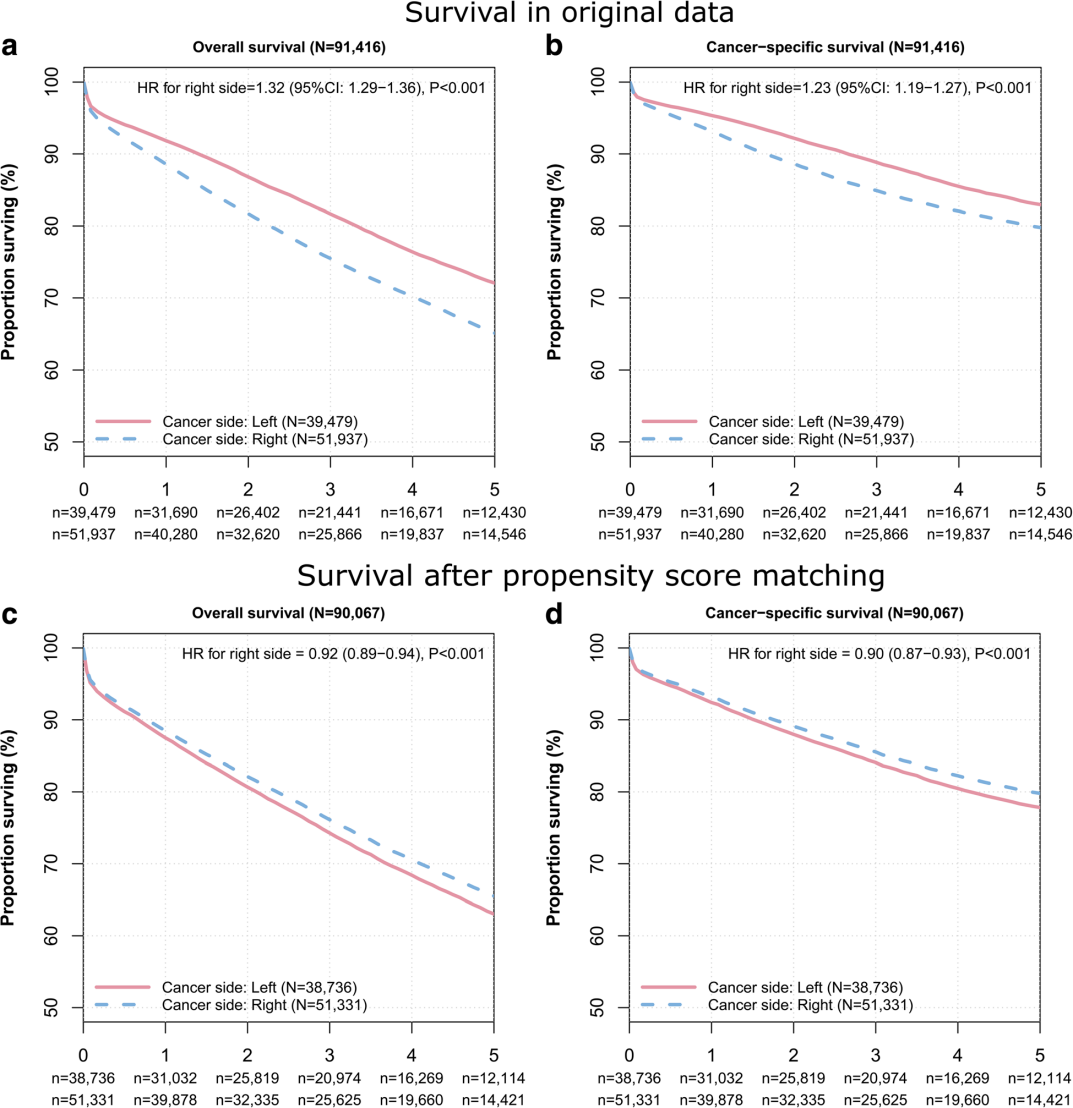
Kaplan-Meier curves for overall and cancer-specific survival. Panel (**a** and **b**) depict the overall and cancer-specific survival in the original data set and panel (**c** and **d**) the overall and cancer-specific survival after propensity score matching. The number of colon cancer patients at risk are given below each plot

The observed 5-year overall survival rate for patients with right-sided colon cancer was 65.1 % (95 % CI: 64.6 to 65.6 %) compared with 72.1 % (95 % CI: 71.5 to 72.6 %) for patients with left-sided colon cancer. The 5-year cancer-specific survival rate for patients with right-sided colon cancer was 79.8 % (95 % CI: 79.4 to 80.2 %) compared with 82.9 (95 % CI: 82.5 to 83.4 %) for patients with left-sided colon cancer (Fig. 2). Table 2 depicts the prognostic value of cancer side and the other patient’s characteristics on adjusted and unadjusted overall and cancer-specific mortality. In unadjusted analysis, right-sided colon cancer was associated with a significantly increased risk of overall mortality (hazard ratio of death [HR] = 1.32, 95 % CI: 1.29 to 1.36, *P* < 0.001) and cancer-specific mortality (HR = 1.26, 95 % CI: 1.21 to 1.30, *P* < 0.001). After multivariable adjustment, patients with right-sided colon cancer had a significantly worse overall (HR = 1.05, 95 % CI: 1.02 to 1.09, *P* < 0.001) but a similar cancer-specific survival (HR = 1.03, 95 % CI: 0.99 to 1.08, *P* = 0.156) compared to patients with left-sided colon cancer. A backward variable selection procedure from the full Cox regression model confirmed a significantly increased risk for overall (HR = 1.05, 95 % CI: 1.02 to 1.09, *P* < 0.001) but not for cancer-specific mortality.

**Table 2:** Prognostic factors for overall and cancer-specific mortality

		Cox regression for overall survival				Cox regression for cancer-specific survival			
		Unadjusted^a^		Adjusted^b^		Unadjusted^a^		Adjusted^b^	
		HR (95 % CI)	*p* ^c^	HR (95 % CI)	*p* ^c^	HR (95 % CI)	*p* ^c^	HR (95 % CI)	*p* ^c^
Cancer side	Left-sided	Reference	<0.001	Reference	0.002	Reference	<0.001	Reference	0.156
	Right-sided	1.32 (1.29–1.36)		1.05 (1.02–1.09)		1.23 (1.19–1.27)		1.03 (0.99–1.08)	
Tumor stage	Stage I	Reference	<0.001	Reference	<0.001	Reference	<0.001	Reference	<0.001
(AJCC 6^th^ ed.)	Stage II	1.65 (1.59–1.71)		1.40 (1.35–1.45)		3.00 (2.81–3.20)		2.47 (2.31–2.65)	
	Stage III	2.34 (2.26–2.42)		2.30 (2.22–2.39)		6.41 (6.02–6.81)		5.79 (5.42–6.18)	
Number of retrieved	<12	Reference	<0.001	Reference	<0.001	Reference	<0.001	Reference	<0.001
Regional lymph	12–16	0.90 (0.87–0.92)		0.82 (0.79–0.84)		0.92 (0.88–0.96)		0.78 (0.75–0.82)	
Nodes	17+	0.75 (0.73–0.77)		0.69 (0.67–0.72)		0.81 (0.78–0.84)		0.66 (0.64–0.69)	
Grading	G1	Reference	<0.001	Reference	<0.001	Reference	<0.001	Reference	<0.001
	G2	1.20 (1.14–1.26)		1.05 (1.00–1.10)		1.46 (1.36–1.56)		1.10 (1.03–1.18)	
	G3	1.76 (1.67–1.85)		1.28 (1.21–1.35)		2.60 (2.41–2.81)		1.51 (1.40–1.64)	
	G4	2.02 (1.83–2.22)		1.50 (1.36–1.66)		2.88 (2.53–3.27)		1.69 (1.49–1.93)	
	Unknown	0.91 (0.84–1.00)		1.04 (0.95–1.14)		1.03 (0.91–1.18)		1.20 (1.06–1.37)	
Histology	Adenocarcinoma	Reference	<0.001	Reference	<0.001	Reference	<0.001	Reference	<0.001
	Adenocarcinoma in adenomatous polyp	0.53 (0.50–0.56)		0.80 (0.76–0.86)		0.36 (0.33–0.40)		0.71 (0.64–0.79)	
	Adenocarcinoma in villous adenoma	0.77 (0.71–0.83)		0.93 (0.86–1.01)		0.62 (0.55–0.70)		0.92 (0.82–1.03)	
	Adenocarcinoma in tubulovillous adenoma	0.63 (0.60–0.67)		0.84 (0.80–0.89)		0.44 (0.40–0.47)		0.73 (0.67–0.79)	
	Mucinous carcinoma	1.12 (1.08–1.17)		1.03 (0.99–1.07)		1.14 (1.08–1.20)		1.05 (0.99–1.10)	
CEA	C0-stage	Reference	<0.001	Reference	<0.001	Reference	<0.001	Reference	<0.001
	C1-stage	1.91 (1.84–1.97)		1.61 (1.56–1.67)		2.29 (2.19–2.40)		1.79 (1.71–1.87)	
	Unknown/Borderline	1.42 (1.38–1.46)		1.37 (1.33–1.41)		1.49 (1.43–1.55)		1.48 (1.42–1.54)	
Operation	Segmental resection	Reference	<0.001	Reference	<0.001	Reference	<0.001	Reference	<0.001
	Hemi/subtotal colectomy	1.27 (1.24–1.30)		1.08 (1.05–1.12)		1.26 (1.22–1.31)		1.11 (1.06–1.16)	
	Other	1.32 (1.18–1.48)		1.34 (1.19–1.50)		1.66 (1.44–1.91)		1.57 (1.36–1.81)	
Year	2004 to 2006	Reference	<0.001	Reference	0.258	Reference	<0.001	Reference	0.542
	2007 to 2009	0.93 (0.91–0.96)		1.02 (0.99–1.05)		0.93 (0.89–0.96)		1.02 (0.98–1.06)	
	2010 to 2012	0.87 (0.83–0.90)		0.98 (0.94–1.03)		0.87 (0.83–0.92)		0.99 (0.94–1.05)	
Age	Up to 49 years	Reference	<0.001	Reference	<0.001	Reference	<0.001	Reference	<0.001
	50 to 64 years	1.14 (1.07–1.22)		1.20 (1.12–1.28)		0.94 (0.87–1.01)		1.04 (0.97–1.13)	
	65 to 79 years	2.18 (2.05–2.33)		2.30 (2.15–2.45)		1.37 (1.28–1.47)		1.57 (1.45–1.69)	
	80+ years	5.15 (4.83–5.49)		5.10 (4.77–5.45)		2.69 (2.50–2.89)		2.88 (2.66–3.11)	
Gender	male	Reference	0.614	Reference	<0.001	Reference	0.001	Reference	<0.001
	female	1.01 (0.98–1.03)		0.75 (0.73–0.77)		1.06 (1.02–1.09)		0.84 (0.81–0.87)	
Ethnicity	Caucasian	Reference	<0.001	Reference	<0.001	Reference	<0.001	Reference	<0.001
	African-American	1.03 (0.99–1.07)		1.17 (1.13–1.22)		1.19 (1.13–1.25)		1.24 (1.18–1.30)	
	Other/Unknown	0.70 (0.67–0.74)		0.77 (0.73–0.81)		0.81 (0.76–0.87)		0.83 (0.78–0.89)	
Marital status	Married	Reference	<0.001	Reference	<0.001	Reference	<0.001	Reference	<0.001
	Single	1.32 (1.27–1.37)		1.46 (1.40–1.52)		1.40 (1.33–1.47)		1.41 (1.33–1.48)	
	Widowed	2.21 (2.15–2.28)		1.42 (1.37–1.46)		1.91 (1.84–1.99)		1.34 (1.28–1.40)	
	Other/Unknown	1.25 (1.20–1.30)		1.28 (1.23–1.33)		1.21 (1.15–1.28)		1.19 (1.13–1.26)	

Hazard ratios (HR) with 95 % confidence intervals (Wald type)

The number of positive regional lymph nodes and T-stage were excluded from analysis to avoid collinearity with stage

*AJCC* American Joint Committee on Cancer, *CEA* Carcinoembryonic antigen

^a^Univariate Cox regression analysis, ^b^multivariable Cox regression analysis full model, ^c^likelihood ratio tests

### Adjusting for patients characteristics with propensity score matching

To further assess whether the colon cancer location has an independent prognostic impact on survival, a bipartite propensity score matching analysis with stratification for stage and year of diagnosis was performed. The rationale for this analysis were the significant imbalances of baseline characteristics in multivariable logistic regression between patients with left- and right-sided colon cancer for all confounders (*P* < 0.001) except for the year of diagnosis (*P* = 0.285) and the marital status (*P* = 0.877, Table 3).

**Table 3:** Imbalances of baseline characteristics between right- and left-sided colon cancer patients

		Logistic regression in raw data (*N* = 91,4166)^a^		Patient characteristics after stratified bipartite propensity score matching (*N* = 90,067)^c^			
		OR (95 % CI)	P^b^	Total	Right-sided carcinoma	Left-sided carcinoma	P^b^
				*N* = 90,067	*N* = 51,331	*N* = 38,736	
Tumor stage	Stage I	D)		24,924 (27.7 %)	13,622 (26.5 %)	11,302 (29.2 %)	E)
(AJCC 6^th^ ed.)	Stage II			33,002 (36.6 %)	19,790 (38.6 %)	13,212 (34.1 %)	
	Stage III			32,141 (35.7 %)	17,919 (34.9 %)	14,222 (36.7 %)	
T-stage	T1	Reference	<0.001	11387.4 (12.6 %)	6378 (12.4 %)	5009.4 (12.9 %)	0.908
	T2	1.45 (1.36–1.55)		17213.6 (19.1 %)	9360 (18.2 %)	7853.6 (20.3 %)	
	T3	1.15 (1.08–1.22)		50984.6 (56.6 %)	29,704 (57.9 %)	21280.6 (54.9 %)	
	T4	1.08 (1.00–1.17)		10481.4 (11.6 %)	5889 (11.5 %)	4592.4 (11.9 %)	
Number of positive	0	Reference	<0.001	57,926 (64.3 %)	33,412 (65.1 %)	24,514 (63.3 %)	0.733
Regional lymph	1	0.82 (0.78–0.87)		10303.2 (11.4 %)	5579 (10.9 %)	4724.2 (12.2 %)	
Nodes	2–3	0.82 (0.77–0.86)		9909.2 (11.0 %)	5631 (11.0 %)	4278.2 (11.0 %)	
	4–6	0.87 (0.81–0.93)		6433.9 (7.1 %)	3650 (7.1 %)	2783.9 (7.2 %)	
	7+	0.94 (0.86–1.01)		5494.7 (6.1 %)	3059 (6.0 %)	2435.7 (6.3 %)	
Number of retrieved	<12	Reference	<0.001	18327.7 (20.3 %)	10,240 (19.9 %)	8087.7 (20.9 %)	0.931
Regional lymph	12–16	1.72 (1.64–1.80)		25,713 (28.5 %)	14,813 (28.9 %)	10,900 (28.1 %)	
Nodes	17+	2.44 (2.33–2.55)		46026.3 (51.1 %)	26,278 (51.2 %)	19748.3 (51.0 %)	
Grading	G1	Reference	<0.001	8274.1 (9.2 %)	4667 (9.1 %)	3607.1 (9.3 %)	0.954
	G2	0.97 (0.91–1.02)		60851.4 (67.6 %)	34,630 (67.5 %)	26221.4 (67.7 %)	
	G3	1.74 (1.62–1.87)		16543.4 (18.4 %)	9599 (18.7 %)	6944.4 (17.9 %)	
	G4	1.92 (1.64–2.24)		1738.2 (1.9 %)	1035 (2.0 %)	703.2 (1.8 %)	
	Unknown	0.92 (0.82–1.03)		2659.8 (3.0 %)	1400 (2.7 %)	1259.8 (3.3 %)	
Histology	Adenocarcinoma	Reference	<0.001	62172.7 (69.0 %)	35,607 (69.4 %)	26565.7 (68.6 %)	0.978
	Adeno-Ca in adenomatous polyp	0.95 (0.89–1.02)		5139.1 (5.7 %)	2883 (5.6 %)	2256.1 (5.8 %)	
	Adeno-Ca in villous adenoma	2.02 (1.82–2.25)		2901.7 (3.2 %)	1664 (3.2 %)	1237.7 (3.2 %)	
	Adeno-Ca in tubulovillous adenoma	1.73 (1.62–1.85)		8333.5 (9.3 %)	4806 (9.4 %)	3527.5 (9.1 %)	
	Mucinous carcinoma	2.23 (2.10–2.37)		11,520 (12.8 %)	6371 (12.4 %)	5149 (13.3 %)	
CEA	C0-stage	Reference	<0.001	31640.5 (35.1 %)	18,476 (36.0 %)	13164.5 (34.0 %)	0.763
	C1-stage	0.88 (0.83–0.92)		17236.6 (19.1 %)	9804 (19.1 %)	7432.6 (19.2 %)	
	Unknown/Borderline	1.00 (0.96–1.04)		41189.8 (45.7 %)	23,051 (44.9 %)	18138.8 (46.8 %)	
Operation	Segmental resection	Reference	<0.001	13557.3 (15.1 %)	7750 (15.1 %)	5807.3 (15.0 %)	0.447
	Hemi/subtotal colectomy	14.3 (13.8–14.8)		75781.8 (84.1 %)	43,171 (84.1 %)	32610.8 (84.2 %)	
	Other	2.66 (2.32–3.05)		727.9 (0.8 %)	410 (0.8 %)	317.9 (0.8 %)	
Year	2004 to 2006	Reference	0.285	31,052 (34.5 %)	17,527 (34.1 %)	13,525 (34.9 %)	E)
	2007 to 2009	0.97 (0.93–1.01)		30,869 (34.3 %)	17,585 (34.3 %)	13,284 (34.3 %)	
	2010 to 2012	0.97 (0.93–1.01)		28,146 (31.3 %)	16,219 (31.6 %)	11,927 (30.8 %)	
Age	Up to 49 years	Reference	<0.001	5545.6 (6.2 %)	3174 (6.2 %)	2371.6 (6.1 %)	0.995
	50 to 64 years	1.56 (1.46–1.66)		21581.8 (24.0 %)	12,332 (24.0 %)	9249.8 (23.9 %)	
	65 to 79 years	2.65 (2.49–2.83)		37577.3 (41.7 %)	21,253 (41.4 %)	16324.3 (42.1 %)	
	80+ years	4.08 (3.80–4.39)		25362.3 (28.2 %)	14,572 (28.4 %)	10790.3 (27.9 %)	
Gender	male	Reference	<0.001	40730.6 (45.2 %)	22,954 (44.7 %)	17776.6 (45.9 %)	0.570
	female	1.30 (1.26–1.35)		49336.4 (54.8 %)	28,377 (55.3 %)	20959.4 (54.1 %)	
Ethnicity	Caucasian	Reference	<0.001	72714.5 (80.7 %)	41,771 (81.4 %)	30943.5 (79.9 %)	0.050
	African-American	1.09 (1.04–1.15)		11763.1 (13.1 %)	6213 (12.1 %)	5550.1 (14.3 %)	
	Other/Unknown	0.66 (0.62–0.70)		5589.4 (6.2 %)	3347 (6.5 %)	2242.4 (5.8 %)	
Marital status	Married	Reference	0.877	47284.7 (52.5 %)	27,005 (52.6 %)	20279.7 (52.4 %)	0.930
	Single	0.98 (0.93–1.03)		10609.8 (11.8 %)	6044 (11.8 %)	4565.8 (11.8 %)	
	Widowed	1.00 (0.95–1.05)		20331.9 (22.6 %)	11,580 (22.6 %)	8751.9 (22.6 %)	
	Other/Unknown	0.99 (0.94–1.05)		11840.6 (13.1 %)	6702 (13.1 %)	5138.6 (13.3 %)	

^a^Multivariable logistic regression with the odds ratio (OR) for right-sided carcinomas in the original raw data set (*N* = 91,416)

*AJCC* American Joint Committee on Cancer, *T-stage* tumor stage, *CEA* Carcinoembryonic antigen

^b^Likelihood ratio tests

^c^After full bipartite propensity score matching with stratification for year of diagnosis and tumor stage, a multivariable conditional logistic regression based on the subgroups obtained by the matching procedure was performed. No significant bias was observed for cancer side

1349 patients were excluded because of lacking counterpart in the other group

Weighted matching causes decimals for the number of patients in the group with left-sided cancer

^D)^Excluded to avoid collinearity with the number of positive regional lymph nodes

^E)^No statistical test because propensity score matching was stratified for year of diagnosis and tumor stage

Before the matching procedure, the propensity score for patients with left-sided colon cancer was 0.346 ± 0.268 compared to 0.739 ± 0.220 in patients with right-sided colon cancer (*P* < 0.001), thus indicating a strong and clinically relevant bias regarding the observed patient and tumor characteristics in the two groups. After the matching procedure, the propensity score was virtually the same in the two groups (0.738 ± 0.220 compared to 0.739 ± 0.220, *P* = 0.622), thus indicating no persisting relevant bias regarding the observed characteristics in the two groups (Table 3). Of the entire cohort, 1349 patients were excluded enabling a propensity score matching in the remaining 90,067 patients. In this cohort, the prognosis of right-sided colon cancer was slightly better compared to left-sided colon cancer regarding overall (HR = 0.92, 95 % CI: 0.89 to 0.94, *P* < 0.001) and cancer-specific mortality (HR = 0.90, 95 % CI: 0.87 − 0.93, *P* < 0.001). The 5-year overall survival rate for patients with right-sided colon cancer was 65.5 % (95 % CI: 65.0 to 66.0 %) compared with 63.0 % (95 % CI: 62.5 to 63.6 %) for patients with left-sided colon cancer. The 5-year cancer-specific survival rate for patients with right-sided colon cancer was 79.8 % (95 % CI: 79.4 to 80.2 %) compared with 77.8 % (95 % CI: 77.3 to 78.3 %) for patients with left-sided colon cancer.

### Stratified stage-wise analysis

**Fig. 3:**
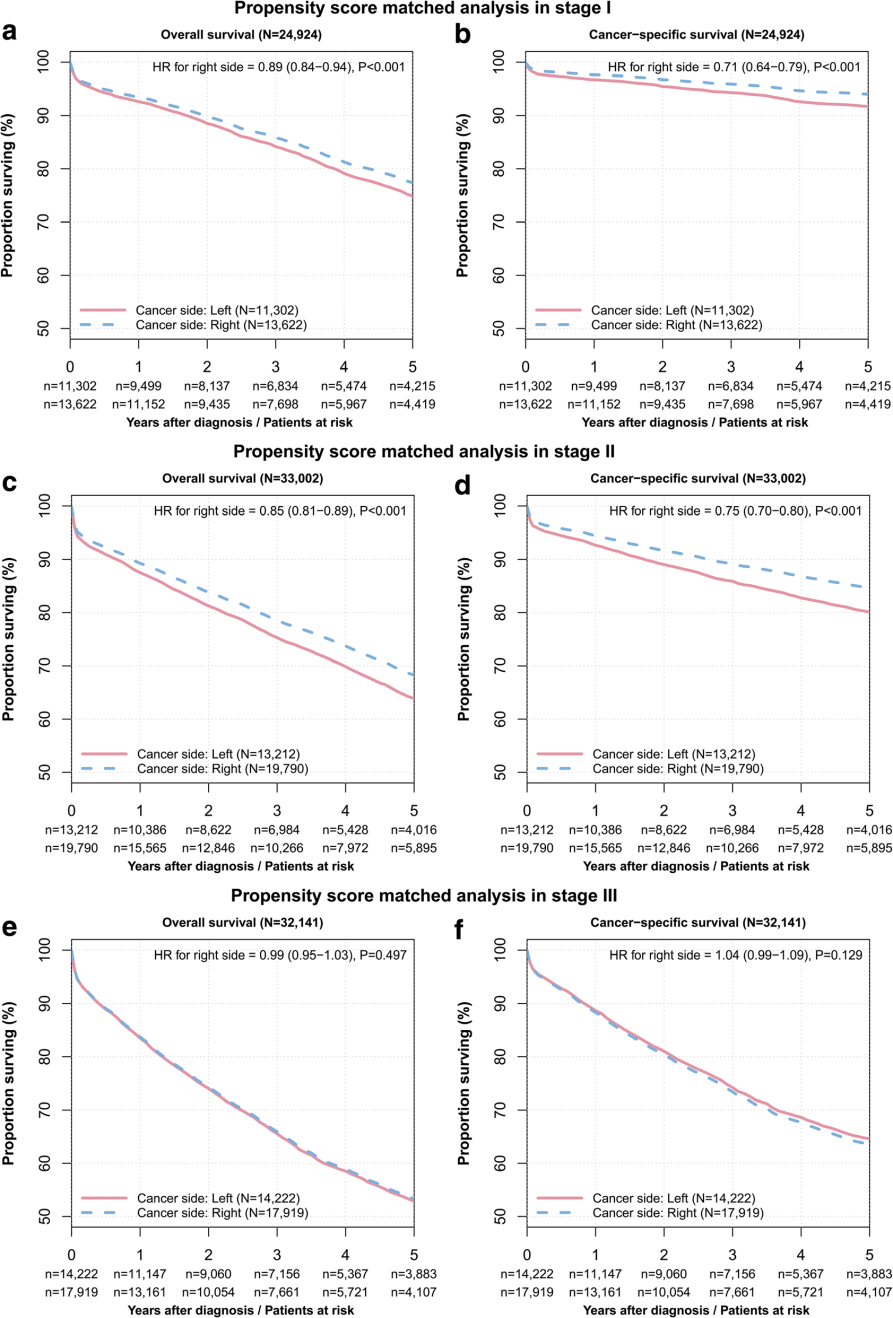
Kaplan-Meier curves for overall and cancer-specific survival after stratified propensity score matching. Panels (**a**, **c**, and **e**) depict the overall survival and panels (**b**, **d**, and **f**) depict the cancer-specific survival for stage I (panels **a** and **b**), stage II (panels **c** and **d**), and stage III (panels **e** and **f**). The number of colon cancer patients at risk are given below each plot

Motivated by an unsuspected better survival in right-sided cancer, a propensity score matched analysis was performed for each stage individually confirming a better prognosis for stage I and II right-sided colon cancer (Fig. 3). In the stage I subgroup, the prognosis of right -sided colon cancer was better regarding overall (HR = 0.89, 95 % CI: 0.84–0.94, *P* < 0.001)) and cancer-specific survival (HR = 0.71, 95 % CI: 0.64–0.79, *P* < 0.001)). In the stage II subgroup, the prognosis of right- sided colon cancer was better regarding overall (HR = 0.85, 95 % CI: 0.81–0.89, *P* < 0.001) and cancer-specific survival (HR = 0.75, 95 % CI: 0.70 to 0.80, *P* < 0.001). In the stage III subgroup, the prognosis of right- and left-sided colon cancer was similar regarding overall (HR = 0.99, 95 % CI: 0.95–1.03, *P* = 0.497) and cancer-specific survival (HR = 1.04, 95 % CI: 0.99–1.09, *P* = 0.129).

The overall five year survival rates for right-sided cancers in stage I, II, and III were 77.4 % (95 % CI: 76.6 to 78.2 %) 68.3 % (95 % CI: 67.6 to 69.1 %), and 53.3 % (95 % CI: 52.5 to 54.2 %) compared to 74.9 % (95 % CI: 74 to 75.8 %), 63.9 % (95 % CI: 63 to 64.8 %), and 52.9 % (95 % CI: 51.9 to 53.8 %) in left-sided carcinomas. The cancer-specific survival rates for right-sided cancers in stage I, II, and III were 94.0 % (95 % CI: 93.6 to 94.5 %), 84.6 % (95 % CI: 84.1 to 85.3 %), and 63.6 % (95 % CI: 62.8 to 64.5 %) compared to 91.7 % (95%CI: 91.1 to 92.3 %), 80.1 % (95 % CI: 79.3 to 80.9 %), and 64.6 % (95 % CI: 63.7 to 65.6 %) in left-sided carcinomas (Fig. 3).

## Discussion

Currently, data regarding the prognosis of right-sided versus left-sided colon cancer are conflicting, however, most studies revealed a poorer survival in right-sided primary tumor location [14–18, 29].

To our knowledge this is the first population-based, propensity score adjusted analysis investigating the prognostic impact of tumor location in non-metastatic colon cancer patients. Being aware of the conflicting data and also of the challenges to handle relevant bias due to substantial imbalances regarding baseline characteristics between right- and left-sided colon cancer patients, we have intentionally selected the propensity score matching as a further statistical method in addition to common multivariate analysis to minimize confounding. In the present analysis, the cohort was partitioned into subgroups containing one or more patients with right-sided colon cancer who were matched to one or more patients with left-sided colon cancer with similar values on the observed covariates in the risk set (bipartite matching). Hence, the main difference between the compared groups in propensity-score matching was the location of the colon cancer (left versus right). The advantage of the propensity score adjusted analysis over multivariate analysis becomes evident from varying results in our study, showing that multivariate analysis is obviously limited in adjusting factors in large retrospective population-based studies.

Based on a large collective of 91,416 patients with stage I - III colon cancer diagnosed between 2004 and 2012, this is the first study providing evidence that the prognosis of patients with right-sided colon cancer in the overall population of stage I – III is similar or even better after adjusting for a strong bias regarding various patient and tumor characteristics by the use of propensity score matching. We thus conclude that the differences in the prognosis between right-sided and left-sided colon cancer patients described in the scientific literature are not real but caused by differences regarding confounders that could not be completely adjusted for in multivariate regression analysis.

Our results differ from the findings published by Weiss et al. [19] demonstrating no 5-year overall survival difference between right- and left-sided colon cancer after adjusting for multiple variables. Weiss et al. [19] analyzed 53,801 colon cancer patients aged 65 years and older from the linked SEER-Medicare database between 1992 and 2005. Interestingly, they found that stage II right-sided colon cancer had lower mortality compared to patients with a left-sided tumor (HR 0.92; 95 % CI, 0.87–0.97, *P* = 0.001), while stage III right-sided colon cancer patients had higher mortality than those with left-sided colon cancer (HR, 1.12; 95 % CI, 1.06 to 1.18; *P* < 0.001) [19]. The present analysis differs from the study by Weiss and colleagues [19] regarding several issues: First, in our investigation patients were included if older than 18 years of age. The majority of patients with colon cancer are usually older persons, however, 37.4 % of all patients in our analysis were younger than 64 years. In our opinion it is key to include younger colon cancer patients as this is a subgroup, which has been shown to dramatically increase in the past decade [30]. Second, we used more recent data from 2004 to 2012 as opposed to the study by Weiss and colleagues (1992–2005) [19]. Finally, while the investigation by Weiss et al. [19] exclusively used conventional multivariable analysis to adjust for imbalances between patients with right - versus left-sided colon cancer, the present investigation used propensity score matching as an additional statistical tool in addition to multivariable analyses.

One limitation of the present study is the lack of information regarding microsatellite instability (MSI) in the SEER database. According to the literature, colon cancer with MSI have a better prognosis [31, 32]. Furthermore, the frequency of MSI is different between right- and left-sided colon cancers and differs also between different tumor stages. One study estimated that the rate of MSI-positive stage II right-sided colon cancers is 25 %, compared to less than 15 % in stage III right-sided cancers [33]. Weiss et al. [19] argued that this difference might contribute to their results showing better prognosis of stage II right-sided cancers. Unfortunately, data on sporadic mismatch repair deficiency, germeline-mutation prompted Lynch Syndrom, BRAF/KRAS/NRAS mutations, or any family history can not be ascertained from the SEER data. Hence, the fraction of patients with these specific subtypes of colon cancer – which are known to vary between the left and right colon side [13]  - are unknown in the present study. However, due to the population-based nature of this analysis  that mirrors the real US population with colon cancer, the lack of this information does not impact our results, albeit limits the extent of interpretation of our data.

Overall, the existing literature shows conflicting data regarding prognosis in right-sided vs. left-sided colon cancer. It is difficult to compare the available studies due to various differences, e.g. in study design and inclusion and exclusion criteria. Suboptimal adjusting of confounders seems to be the major difficulty to obtain a thorough analysis regarding the prognostic impact of tumor location, pointing out the strength of our study with further adjustment by propensity score matching. Our investigation provides evidence that right-sided tumour location itself is associated with improved prognosis compared to the left-sided colon cancer, mostly due to better prognosis in stage I and stage II patients.

We would like to acknowledge the limitations of the present study. First, information regarding chemotherapeutic treatments of patients can not be ascertained from the SEER data. Second, information on comorbidities, performance status and data regarding family history are also not available in the SEER database. To which extent these parameters might have influenced the analysis remains unclear. Third, while we did risk-adjust for known confounders, potential bias due to unknown confounding cannot be excluded. Unobserved confounders represent a relevant limitation to the generalizability of the propensity score method. Finally, although propensity-score adjustment represents an additional, valuable statistical tool used in the present analyses, the results must be interpreted with some caution due to a fraction of unmatched patients.

## Conclusions

This population-based propensity score adjusted analysis on stage I - III colon cancer provides evidence that the prognosis of patients with right-sided colon cancer is better compared to left-sided colon cancer mostly due to a better survival in stage I and stage II patients. This finding questions the paradigm of worse survival in right-sided colon cancer patients claimed by the majority of previous research.

## Abbreviations

AJCC, American Joint Committee on Cancer; CEA, carcinoembryonic antigen; CI, confidence interval; HR, Hazard Ratio; IQR, interquartile ratio; SEER, Surveillance, Epidemiology, and End Results; T-stage, tumor stage

## References

[1] Jemal A, Bray F, Center MM, Ferlay J, Ward E, Forman D (2011). Global cancer statistics. CA Cancer J Clin.

[2] Brenner H, Kloor M, Pox CP (2014). Colorectal cancer. Lancet.

[3] Cucino C, Buchner AM, Sonnenberg A (2002). Continued rightward shift of colorectal cancer. Dis Colon Rectum.

[4] Saltzstein SL, Behling CA (2007). Age and time as factors in the left-to-right shift of the subsite of colorectal adenocarcinoma: a study of 213,383 cases from the California Cancer Registry. J Clin Gastroenterol.

[5] Hansen IO, Jess P (2012). Possible better long-term survival in left versus right-sided colon cancer - a systematic review. Dan Med J.

[6] Iacopetta B (2002). Are there two sides to colorectal cancer?. Int J Cancer.

[7] Nawa T, Kato J, Kawamoto H, Okada H, Yamamoto H, Kohno H (2008). Differences between right- and left-sided colon cancer in patient characteristics, cancer morphology and histology. J Gastroenterol Hepatol.

[8] Maruta M, Kotake K, Maeda K (2007). Colorectal cancer in Japan. Rozhl Chir.

[9] Papagiorgis P, Oikonomakis I, Karapanagiotou I, Wexner SD, Nikiteas N (2006). The impact of tumor location on the histopathologic expression of colorectal cancer. J BUON.

[10] Geelhoed GW, Crossland SG (1981). Carcinoma of the right colon: a change in characteristic configuration?. South Med J.

[11] Elnatan J, Goh HS, Smith DR (1996). C-KI-RAS activation and the biological behaviour of proximal and distal colonic adenocarcinomas. Eur J Cancer.

[12] Lanza G, Maestri I, Ballotta MR, Dubini A, Cavazzini L (1994). Relationship of nuclear DNA content to clinicopathologic features in colorectal cancer. Mod Pathol.

[13] Sinicrope FA, Shi Q, Smyrk TC, Thibodeau SN, Dienstmann R, Guinney J (2015). Molecular markers identify subtypes of stage III colon cancer associated with patient outcomes. Gastroenterology.

[14] Benedix F, Kube R, Meyer F, Schmidt U, Gastinger I, Lippert H, Colon/Rectum Carcinomas (Primary Tumor) Study Group (2010). Comparison of 17,641 patients with right- and left-sided colon cancer: differences in epidemiology, perioperative course, histology, and survival. Dis Colon Rectum.

[15] Meguid RA, Slidell MB, Wolfgang CL, Chang DC, Ahuja N (2008). Is there a difference in survival between right- versus left-sided colon cancers?. Ann Surg Oncol.

[16] Suttie SA, Shaikh I, Mullen R, Amin AI, Daniel T, Yalamarthi S (2011). Outcome of right-and left-sided colonic and rectal cancer following surgical resection. Colorectal Dis.

[17] Faivre-Finn C, Bouvier-Benhamiche AM, Phelip JM, Manfredi S, Dancourt V, Faivre J (2002). Colon cancer in France: evidence for improvement in management and survival. Gut.

[18] Derwinger K, Gustavsson B (2011). Variations in demography and prognosis by colon cancer location. Anticancer Res.

[19] Weiss JM, Pfau PR, O’Connor ES, King J, LoConte N, Kennedy G (2011). Mortality by stage for right- versus left-sided colon cancer: analysis of surveillance, epidemiology, and end results—Medicare data. J Clin Oncol.

[20] Surveillance, Epidemiology, and End Results (SEER) Program (www.seer.cancer.gov) Research Data (1973-2012), National Cancer Institute, DCCPS, Surveillance Research Program, Surveillance Systems Branch, released April 2015, based on the November 2014 submission. www.seer.cancer.gov. Accessed 15 April 2015.

[21] Wingo PA, Jamison PM, Hiatt RA, Weir HK, Garguillo PM, Hutton M (2003). Building the infrastructure for nationwide cancer surveillance and control--a comparison between the National Program of Cancer Registries (NPCR) and the Surveillance, Epidemiology, and End Results (SEER) Program (United States). Cancer Causes Control.

[22] Fritz A, Percy C, Jack A, Shanmugaratnam K, Sobin L, Parkin DM (2000). International classification of disease for oncology.

[23] Grambsch PM, Therneau TM (1994). Proportional hazards tests and diagnostics based on weighted residuals. Biometrika.

[24] Joffe MM, Rosenbaum PR (1999). Invited commentary: propensity scores. Am J Epidemiol.

[25] Rosenbaum PR (1987). Model-based direct adjustment. J Am Stat Assoc.

[26] Rubin DB (1997). Estimating causal effects from large data sets using propensity scores. Ann Intern Med.

[27] Ho D, Imai K, King G, Stuart E (2007). Matching as nonparametric preprocessing for reducing model dependence in parametric causal inference. Polit Anal.

[28] Hansen BB, Klopfer SO (2006). Optimal full matching and related designs via network flows. J Comput Graph Stat.

[29] Jess P, Hansen IO, Gamborg M, Jess T, on behalf of the Danish Colorectal Cancer Group (2013). A nationwide Danish cohort study challenging the categorisation into right-sided and left-sided colon cancer. BMJ Open.

[30] Bailey CE, Hu CY, You YN, Bednarski BK, Rodriguez-Bigas MA, Skibber JM (2015). Increasing disparities in the age-related incidences of colon and rectal cancers in the United States, 1975-2010. JAMA Surg.

[31] Popat S, Hubner R, Houlston RS (2005). Systematic review of microsatellite instability and colorectal cancer prognosis. J Clin Oncol.

[32] Malesci A, Laghi L, Bianchi P, Delconte G, Randolph A, Torri V (2007). Reduced likelihood of metastases in patients with microsatellite-unstable colorectal cancer. Clin Cancer Res.

[33] Jernvall P, Mäkinen MJ, Karttunen TJ, Mäkelä J, Vihko P (1999). Microsatellite instability: impact on cancer progression in proximal and distal colorectal cancers. Eur J Cancer.

